# PSMA response evaluation in follow-up PSMA-PET/CT after stereotactic ablative body radiotherapy (SABR) for oligometastases in prostate cancer

**DOI:** 10.1016/j.ctro.2025.101021

**Published:** 2025-07-23

**Authors:** Anna-Lena Zang, Timo Maier, Sandra Freitag-Wolf, Alexander Fabian, Severin Rodler, Jürgen Dunst, David Krug, Ulf Lützen, Olaf Wittenstein

**Affiliations:** a Department of Radiotherapy, University Hospital Schleswig-Holstein, Campus Kiel, Arnold-Heller-Str. 3, 24105 Kiel, Germany; b Department of Nuclear Medicine, University Hospital Schleswig-Holstein, Campus Kiel, Arnold-Heller-Str. 3, 24105 Kiel, Germany; c Institute for Medical Informatics and Statistics, Christian-Albrechts University Kiel, Brunswikerstr. 10, 24105 Kiel, Germany; d Department of Radiotherapy and Radiation Oncology, University Hospital Hamburg-Eppendorf, Martinistr. 52, 20251 Hamburg, Germany; e Department of Urology, University Hospital Schleswig-Holstein, Campus Kiel, Arnold-Heller-Str. 3, 24105 Kiel, Germany

**Keywords:** Metastasis-directed therapy, Oligometastasic prostate cancer, Stereotactic body radiotherapy, Prostate specific antigen, PSMA-response, PSMA PET/CT

## Abstract

**Purpose:**

PSMA-PET/CT is frequently used for staging patients with de-novo or recurrent prostate cancer (PCa). In patients with oligometastatic PCa PSMA-PET/CT guided stereotactic ablative body radiotherapy (SABR) is a common treatment option. Follow-up is regularly performed via measurement of prostate-specific-antigen (PSA) level. Response assessment based on follow-up PSMA-PET/CTs is poorly studied. Therefore, we report on long-term local tumor response using repeated PSMA-PET/CTs of patients with oligometastatic PCa after PSMA-PET/CT guided SABR.

**Methods/Patients:**

Patients with de-novo oligometastatic or oligoprogressive PCa who received PSMA-PET/CT-directed SABR with 5 × 7 Gy of at least one bone or lymph node lesion between 2015 and 2019 and had one or more follow-up PSMA-PET/CT were included in this retrospective single center analysis. PSMA response was evaluated by visual and quantitative assessment of local PSMA uptake pre- and post-SABR.

**Results:**

Overall, 48 patients with 97 irradiated lesions and a total of 145 PSMA-PET/CT-scans were analyzed. 26 patients received androgen-deprivation-therapy (ADT) at any time. Median SUV_max_ per lesion was 10.88 (range 1.59–122.11) before SABR with a median CTV of 4.75 cm^3^ (Range 0.68–60.4 cm^3^). In the first follow-up PET/CT after a median of 13 months (range 3–42) after SABR, median SUV_max_ per lesion declined to 2.2 (range 0.13–26.09). Complete remission (CR) was observed in 49 lesions, partial remission in 32 and stable disease in 12 lesions. Four lesions were non-responders. Over the course of up to five follow-up PSMA-PET/CTs a maximum of 90 % of the lesions showed CR. Median time to SUV_min_ was 19 months (range 3–50). 5-year local control was 86 %. No short-term or long-term toxicities were reported.

**Conclusion:**

PSMA-PET/CT directed SABR provides excellent long-term local tumor control of 90% in bone and lymph node metastasis of oligometastatic PCa and is well tolerated. PSMA activity may further decrease after initial re-imaging with PSMA-PET/CT.

## Highlights

• 97 PSMA-PET-positive lesions in 48 patients with oligometastatic prostate cancer were treated with 35 Gy in 5 fractions.
• The five-year local control rate was high (86 %).
• PSMA-PET expression in irradiated lesions decreases slowly over time (in median 19 months to SUV_min_).

## Introduction

Oligometastatic disease has been regarded as a paradigm shift for several years. Under certain conditions, oligometastatic patients can benefit from additive local therapy. [1,2] In metastatic prostate cancer, radiotherapy of the prostate can prolong survival in patients with de-novo diagnosis of low volume metastatic disease. [3] Moreover, in patients with a limited number of metastatic lesions, stereotactic ablative body radiotherapy (SABR) as a non-invasive metastasis-directed therapy (MDT) can improve the freedom from recurrence and extend the interval until the start of androgen-deprivation therapy. [1,2,[4], [5], [6], [7], [8]] MDT has achieved promising results in freedom from biochemical recurrence in several studies, e.g. with a hazard ratio of around 0.30 in the randomized ORIOLE study. [4] However, ORIOLE and other studies were too small and not designed to detect a survival benefit, and the systemic therapy was ADT (androgen-deprivation therapy) or no systemic therapy according to the standard of care at that time. At the same time, a clear survival benefit was demonstrated by the addition of the new hormonal agents to ADT. [9] This modern systemic therapy is therefore considered as the contemporary standard of care and treatment option of first choice.

In studies with ablative radiotherapy of oligometastases, biochemical progression and imaging-detected progression were the main endpoints used. Imaging (e.g. in the ORIOLE study) included conventional procedures, i.e. Computer Tomography (CT), Magnet Resonance Imaging (MRI) and bone scintigraphy. Positron emission tomography CT (PET/CT) with radiolabeled ligand against prostate-specific membrane antigen (PSMA) which shows superior sensitivity and specificity compared to other imaging modalities even at low PSA-levels [[10], [11], [12], [13], [14], [15], [16]], was not routinely used for primary diagnosis or follow-up. Furthermore, PSMA-PET/CT information can be used to improve target volume definition in radiotherapy and treat even small and, with conventional imaging, almost undetectable lesions with minimal toxicity. [1,17,18] A subgroup analysis of the ORIOLE-trial suggested that targeting of all PSMA-positive metastases results in superior progression free and distant metastases free survival. [4].

After MDT, monitoring of PSA-level is used for further treatment decisions including administration of repeated PSMA-PET/CT scans in case of rising PSA. Correct interpretation of the PSMA expression in irradiated and possibly new detectable non-irradiated lesions is crucial for further treatment decision (repeat MDT with irradiation of new lesions and/or re-irradiation of non-responding lesions vs. initiation of ADT). Our hypothesis was therefore to use further PSMA-PET/CTs performed after PSMA-PET/CT guided ablative radiotherapy to evaluate local treatment success, assuming that PSMA remission on subsequent PSMA-PET imaging can be used as a marker for local tumor control. An evaluation of the first five patients with 18 metastatic bone lesions had shown a favorable PSMA response rate of 88 % after one year. [19] Other studies have meanwhile supported these findings. [[20], [21], [22]] The objective of this current analysis was to evaluate PSMA response in a larger number of patients with long term follow-up.

## Materials and methods

### Study objective

The retrospective single center analysis was conducted with the aim to determine PSMA response in follow-up PSMA-PET/CTs in patients with de novo or metastatic prostate cancer who had undergone SABR for PSMA-PET/CT detected bone and lymph node oligometastases. The study was approved by the ethical committee of the Medical Faculty of the Christian-Albrechts-University Kiel (D 545/19).

### Patient population

The analysis included men with the following inclusion criteria: de-novo diagnosis of oligometastatic disease or oligoprogression detected with PSMA-PET/CT in the period from May 2014 through September 2019, SABR with 5 fractions of 7 Gy to at least one bone or lymph node metastasis and at least one additional PSMA-PET/CT during follow-up.

### PSMA PET/CT imaging

Generator-derived ^68^Ga was labelled with PSMA-11 and administered through intravenous injection. The average activity injected was 134 MBq (range 73-282; calculated from 2.5 MBq/kg body weight). Time delay between administration of the tracer and image acquisition was 64 min (range 44-177). For image acquisition, a Biograph mCT 40 (Siemens, Erlangen, Germany) was utilized. Scans were collected from the vertex/or skull base to the upper thigh. Auxiliary low-dose CT was acquired for attenuation correction and anatomic correlation. Images were interpreted in consensus by a nuclear medicine physician and a dual specialist in nuclear medicine and radiology. Pathologically increased PSMA-expression in metastatic lesions was visually identified and maximal standardized uptake value (SUV_max_) was measured. Follow-up scans were performed due to further biochemical recurrence or to assess the option for PSMA-targeted radioligand therapy (PRLT). A routine check in the form of a PSMA-PET/CT examination was not carried out for reasons of radiation protection.

### Treatment and delivery

A CT with contiguous 3.0 mm slice thickness was performed for treatment planning and the initial PSMA-PET scans were co-registered with the planning CTs. All treatments were performed with a TrueBeam STx treatment system (Varian, Paolo Alto, US) within 6 months after the initial PSMA-PET-scan. The gross tumor volume (GTV) was defined as the PSMA-PET-positive lesion after visual assessment with inclusion of osteoblastic changes. The GTV was expanded by 2-3 mm to create the clinical target volume (CTV) (Suppl. Fig. 1). Typically, no additional elective nodal or bone regions were included in the CTV. The CTV was expanded by another 2-3 mm to account for setup uncertainties to create the planning target volume (PTV). A cone-beam-CT was performed before each treatment fraction. Radiation dose was 35 Gy in 5 fractions (PTV D_50%_). D95% had to be at least 33.25 Gy. All plans used volumetric modulated arc therapy with at least two coplanar arcs and fulfilled the institutional OAR dose constraints.

### PSMA response assessment

Post-SABR PSMA-PET/CT scans were compared to all prior PSMA-PET/CT scans and SUV_max_ of every irradiated bone or lymph node metastasis was measured. PSMA response on PET scans was assessed using criteria published by Fanti et al. [23], categorizing lesions into responders (R) and non-responders (NR). Among R we identified complete response (CR, no pathological PSMA-expression within the metastatic lesion), partial response (PR, reduction of SUV_max_ > 30 % compared to pre-SABR SUV_max_ but still pathologically increased) − and stable disease (SD, SUV_max_ reduction or elevation <30 %). NR were defined by SUV_max_ elevation >30 % in the post-SABR PSMA-PET/CT imaging.

### Toxicity criteria

According to German radiation protection law, all patients had at least one follow-up visit 6 weeks after treatment for evaluation of short-term toxicities and most patients were followed thereafter in yearly intervals. The toxicity ranking was based on CTCAE v5 criteria. [24] Long-term toxicities were also documented irregularly when patients were presented in our institution in case of biochemical recurrence or for discussing results of follow-up PSMA-PET/CT scans.

### Statistical analysis

Data were analyzed using SPSS (IBM, Armonk, US) and R (R Foundation for Statistical Computing, Vienna, Austria). For the descriptive statistics medians were calculated for continuous, non-normally distributed variables. The Shapiro-Wilk test was used to check the normal distribution of variables.

In the bivariate analysis of the influence of the PSMA-PET/CT interval from baseline to the first follow-up PSMA-PET/CT on the SUV_max_ value, the Spearman rank correlation coefficient was determined in the presence of non-normally distributed variables. There was a two-tailed significance level of α = 0.05. The Kaplan-Meier estimator was used to represent the local control rate of the irradiated lesions. A progression from prior SBRT PET to follow-up PSMA-PET/CT was defined as an event (SUV_max_ increase ≥30 %) and the time of occurrence was determined in months. Lesions were censored after their last PSMA-PET/CT without further follow-up PSMA-PET/CT.

## Results

### Patients’ characteristics

From May 2014 through September 2019 a total of 109 patients with de-novo diagnosis of oligometastatic PCa or diagnosis of oligoprogressive PCa were treated with PSMA-PET/CT-guided radiotherapy. Of these, 61 patients were excluded because they had no follow-up PSMA-PET/CT (n = 8) or because they received radiation treatment which did not fit the inclusion criteria (n = 53); this latter group consisted of patients with local and/or regional and/or paraaortic lymph node metastases who were treated with either salvage radiotherapy to the prostatic bed or to elective lymph node areas. The remaining 48 patients with bone and lymph node metastases who received MDT with 5 × 7 Gy were included in this analysis.

The mean patients’ PSA-level prior to SABR was 2.01 ng/ml [range 0.03-168.0 ng/ml]. Further patients’ characteristics are listed in Table 1. The median follow-up period at the time of analysis was 11 months (range 3–42 months).

**Table 1:** Patient characteristics before SABR / baseline-PSMA-PET/CT.

Number of patients	48
Age [years] at 1st PSMA-PET/CT median (range)	72 (48–83)

D’Amico risk group at primary diagnosis	

Low Risk	0
Intermediate Risk	4
High Risk	16
Node positive	19
no sufficient data	9

ADT	

ADT before/after SABR	26
no ADT/no data	22

Prior therapy before SABR	

Prostatectomy	24
Prostatectomy + Radiotherapy (adjuvant/salvage)	20
Radiotherapy	0
No prior therapy	4

*Abbreviations*: ADT = Androgen-Deprivation-Therapy; CT = Computer Tomography; PET = Positron-Emission-Tomography; PSMA = Prostate-Specific-Membrane-Antigen; SABR = Stereotactic Ablative Radiotherapy.

### Radiation treatment and systemic therapy

A total of 30 patients with 67 bone lesions and 18 patients with 30 lymph node lesions were treated with SABR consisting of 5 × 7 Gy to all detected lesions (mean 2.02 treated lesions per patient). Overall 26 patients received ADT alone or in combination with a new antihormonal agent at any time during follow-up. Mean GTV and CTV were 1.8 cm^3^ and 4.75 cm^3^, respectively (range 0.14-23.9 cm^3^ and 0.68-60.4 cm^3^, respectively). There was no documented acute or late toxicity ≥grade 2.

### Follow-up PSMA-PET/CTs

Every patient had at least one additional PSMA-PET/CT-scan after SABR. In summary, 145 PSMA-PET/CT-scans were analyzed. Response of all 97 treated lesions was examined in a subsequent PSMA-PET/CT after a median follow-up of 13 months (range 3-42 months). 62 lesions were evaluated in a second follow-up PSMA-PET/CT after a median of further 11 months (range 4-33 months). A total of 30 lesions were evaluated with a third PSMA-PET/CT after in median further 11 months (range 6-18 months) and 18 lesions with a fourth follow-up PSMA-PET/CT after a median of further 10 months (range 4-29 months) (Table 2).

**Table 2:** Detailed PSA-level and PSMA-PET/CT characteristics of patients (n = 48) receiving SABR to bone oligometastases (n = 97).

PSA-level [ng/ml]	median (range)
at baseline- PSMA-PET/CT (*n* = 48)	2.01 (0.03–168.0)
at 1st FU-PSMA-PET/CT (*n* = 48)	3.5 (0.03–914.0)
at 2nd FU-PSMA-PET/CT (*n* = 19)	4.19 (0.5–99.6)
at 3rd FU-PSMA-PET/CT (*n* = 11)	3.53 (0.27–83.0)
at 4th FU-PSMA-PET/CT (*n* = 7)	4.19 (0.84–38.8)
at 5th FU-PSMA-PET/CT (*n* = 4)	13.85 (1.41–22.2)
at 6th FU-PSMA-PET/CT (*n* = 2)	26.05 (10.1–42.0)

Interval scan to scan [months]	median (range)

Baseline to 1st FU- PSMA-PET/CT	13 (3–42)
1st to 2nd FU-PSMA-PET/CT	11 (4–33)
2nd to 3rd FU-PSMA-PET/CT	11 (6–18)
3rd to 4th FU-PSMA-PET/CT	10 (4–29)
4th to 5th FU-PSMA-PET/CT	9 (6–12)
5th to 6th FU-PSMA-PET/CT	13 (8–17)

Patient́s follow-up period [months] median (range)	11 (3-42)

Mean analyzed lesions per patient	2.02

Total number of analyzed PSMA-PET/CTs	145

SUV_max_ (n = analyzed lesions)	median (range)

Baseline-PSMA-PET/CT (n = 97)	10.88 (1.59–122.11)
1st FU-PSMA-PET/CT (n = 97)	2.2 (0.13–26.09)
2nd FU-PSMA-PET/CT (n = 62)	1.48 (0.41–71.25)
3rd FU-PSMA-PET/CT (n = 30)	1.29 (0.6–9.08)
4th FU-PSMA-PET/CT (n = 18)	1.38 (0.59–3.25)
5th FU-PSMA-PET/CT (n = 5)	1.06 (1.05–1.99)
6th FU-PSMA-PET/CT (n = 3)	0.91 (0.88–0.94)

Time to SUV_min_ [months] median (range)	19 (3-50)

GTV [cm^3^]	median (range)

all lesions (n = 97)	1.82 (0.14–23.9)
bone lesions (n = 67)	2.03 (0.14–23.9)
lymph node lesions (n = 30)	1.37 (0.18–13.61)

CTV [cm^3^]	median (range)

all lesions (n = 97)	4.75 (0.68–60.4)
bone metastases (n = 30)	4.83 (0.68–60.4)
lymph node metastases (n = 30)	3.94 (0.72–24.72)

*Abbreviations*: CT = Computer Tomography; CTV = Clinical Target Volume; GTV = Gross Tumor Volume; FU-PSMA-PET/CT = Follow-Up PSMA-PET/CT-scan; PSA = Prostate-Specific-Antigen; SUV_min_ = minimum Standardized-Uptake-Value; SUV_max_ = maximum Standardized-Uptake-Value; GTV = Gross Tumor Volume; CTV = Clinical Target Volume.

### PSMA response

**Fig. 1:**
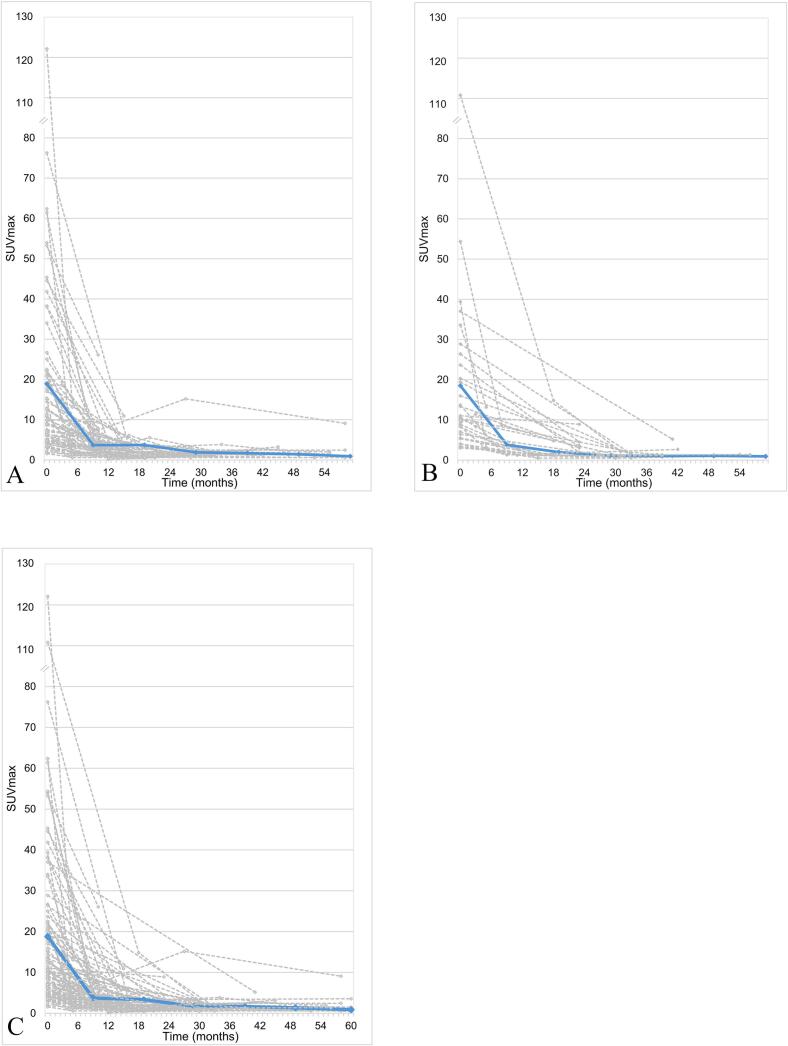
Fitting curves showing the course of maximum Standard Uptake Value levels (SUV_max_) in PSMA-PET/CT of every single bone (n = 67) (a), lymph node (n = 30) (b) and combined (c) lesions in the course after stereotactic ablative radiotherapy to 97 lesions of 48 patients with prostate cancer (mean values in blue). (For interpretation of the references to colour in this figure legend, the reader is referred to the web version of this article.)

**Fig. 2:**
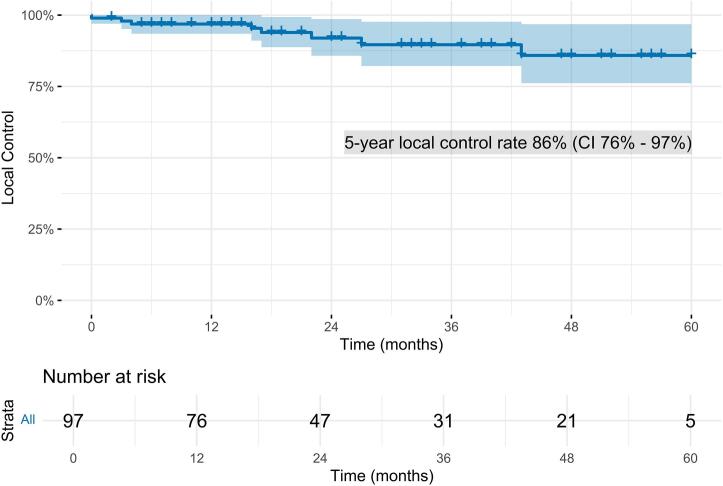
Kaplan-Meier plot of local control rate of bone and lymph node oligometastases (n = 97) after stereotactic ablative radiotherapy of patients with prostate cancer (n = 48) based on multiple PSMA-PET/CT scans during follow-up and evaluation according to PSMA PET/CT response assessment criteria in prostate cancer proposed by Fanti et al. [23] A significantly progressive PSMA expression of the target lesion from one scan to the next scan quantified by SUV_max_ counted as event. The median follow-up time was 11 months.

**Fig. 3:**
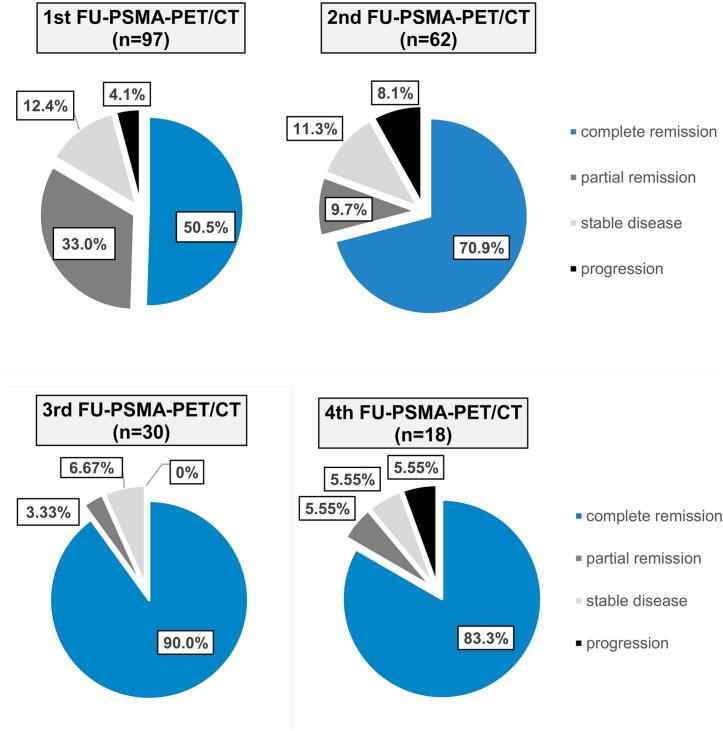
PSMA-response assessment of PSMA-PET/CT-positive lesions treated with SABR according to PSMA-PET/CT response assessment criteria in prostate cancer proposed by Fanti et al. [23] SUV_max_ of every lesion in every follow-up-PSMA-PET/CT (FU-PSMA-PET/CT) was correlated with SUV_max_ in the immediately preceding PSMA-PET/CT-scan and the lesions were distributed to one of the four response groups. The pie charts show distribution pattern to response groups in percentage. All FU-PSMA-PET/CTs were not regularly performed but triggered by biochemical recurrence of the underlying disease.

The mean SUV_max_ of the single lesions before SABR was 10.88 [range 1.59-122.11]. SUV_max_ of irradiated lesions decreased significantly after SABR in the course of multiple PSMA-PET/CT-scans as shown in Fig. 1 with a two-sided significance of *p* = 0.001 and a spearman-rho coefficient of -0.563 (95 % CI -0.692 to -0.399). Median SUV_max_ after irradiation were 2.2, 1.48, 1.29 and 1.38, respectively, in the 1st, 2nd, 3rd and 4th follow-up PSMA-PET/CT (Tab. 2). Median time to SUV_min_ was 19 months (range 3-50). The 5-year local control of all irradiated initial PSMA-PET/CT positive lesions was 86 % (CI 76 %-97 %) (Fig. 2). The number of lesions categorized as complete responders (CR) increased over time. After the first follow-up PSMA-PET/CT, 12 lesions (12.4 %) were SD, 32 (33.0) PR, 49 (50.5 %) CR and 4 (4.1 %) NR compared to the baseline PSMA-PET/CT-scan before SBRT. After the second follow-up PSMA-PET/CT compared to the first follow-up PSMA-PET/CT allocation was as follows: 70.9 % CR, 9.7 % PR, 11.3 % SD and 8.1 % NR (progression). Detailed response categorization is shown in Fig. 3.

## Discussion

Several studies have used PSMA-PET/CT for response evaluation after radiotherapy for prostate cancer oligometastases. [[19], [20], [21],^25^] Including 48 patients with 97 bone and lymph node lesions our cohort belongs to one of the largest relatively homogenous cohorts of metastatic prostate cancer patients who were (a) treated with ablative radiation doses to PSMA-PET/CT-detected oligometastases and (b) had a functional response assessment with multiple PSMA-PET/CT-scans. Hotta et al. recently published a larger cohort of 89 patients with 217 lesions [25] while Sheikh et al. published a series with 32 patients with 49 lesions. [26] Our data supports their findings and demonstrate the high local efficacy of radiotherapy and the reliability of PSMA-PET/CT in this setting. The main findings of our study are:

1. A decrease in PSMA expression and long-term PSMA control were observed in the vast majority of lesions. The PSMA control rate of 86 % after 5 years compares favorably with the expected long-term local control after SABR for oligometastases. [1,2,6,7]
2. For evaluation of PSMA response, we used the criteria that have recently been published by Fanti and coworkers. [23] These criteria are based only on the agreement of a panel of international experts and they have so far not been evaluated in prospective trials. Nevertheless, our study supports the clinical requirement for these criteria. Further evaluation with inclusion of PSMA kinetics is needed.
3. We observed a decreasing PSMA expression of metastatic lesions after SABR. However, the velocity of decrease was slow. In our cohort the maximum effect of SABR with lowest SUV_max_ was observed in median 19 months after SABR (time to SUV_min_). This is even slower than in the series of Hotta et al. who observed the nadir in PSMA uptake after 9 to 12 months. [25] Considering this kinetics of PSMA expression is very important for the interpretation of follow-up PSMA-PET/CTs, especially in patients who have a new PSMA-PET/CT for rising PSA-level after SABR for oligometastases. Based on our data, persistent PSMA expression in one or more treated lesions should be considered as a contraindication for MDT to, new lesion in patients with a rising PSA-level. Correct interpretation of these findings is necessary and helpful especially if SABR is used to delay the initiation of ADT. The decrease in PSMA expression observed in this study resembles the decline of PSA-level after radiotherapy with expanded curative intent for oligometastastic localized prostate cancer. [25,27] This suggests that the biological basis for response to radiotherapy is similar in the primary tumor and in oligometastases.
4. The fractionation regimen in our study (35 Gy in 5 fractions) was based on the HYPOSTAT-regimen used for treatment of localized prostate cancer and it is comparable to regimens in other larger studies, e.g. HYPO-RT-PC and the PACE-A and B studies. [[28], [29], [30], [31]] The observed local control in oligometastases (bone and lymph node lesions) is similar to the control rate for the primary tumor. This suggests that oligometastases are comparable to the primary tumor with regard to radiation sensitivity.
5. Our target volume definition of the GTV for bone and lymph node lesions was strictly based on the PSMA-PET/CT detected PSMA expression with only a geometric CTV expansion of 2–3 mm but no further inclusion of elective anatomical regions which might have been considered at high risk for microscopic disease. For both, radiation of PSMA-PET/CT-positive lymph nodes or bone metastases, the most effective target volume definition remains unclear. Further studies like PEACE-V trial for elective lymph node irradiation versus MDT only approach [32] are currently investigating if elective nodal irradiation improves outcome in nodal oligometastases. Our results suggest that PSMA-PET/CT-guided SABR with minimal margin expansion delivers high local control in bony and nodal oligometastases.

Our study, on the other hand, has some limitations. First, follow-up PSMA-PET/CTs were not systematically performed for response evaluation but were carried out only in case of rising PSA-level. This is related to radiation protection legislation in Germany. Thus, the intervals between PSMA-PET/CTs showed relatively large inter- and intraindividual differences. Therefore, the impact of patient and tumor related characteristics (e.g. Gleason score) on response rate and response dynamics cannot be clearly estimated.

Secondly, due to this selection bias, some patients probably have been missing because they either had ongoing biochemical control after SABR and therefore no indication for a new PSMA-PET/CT or because they presented with clinical or radiographic progression which made additional PSMA-PET/CT imaging redundant. Both factors might, at least theoretically, impact on the observed PSMA response rate with either underestimation or overestimation of remission rates.

Thirdly, a significant limitation of our study is the fact that systemic therapy was not specified, and we did not have detailed information on previous systemic therapy at the time of follow-up PET-CTs in all patients. However, the rising PSA value rules out effective systemic therapy at the time of the examination. The fact that follow-up PSMA-PET/CTs usually found new metastases with metabolic control of the metastases which received SABR is, in our view, indirect evidence of the effectiveness of radiotherapy and excludes a significant contribution of systemic therapy to local tumor control of irradiated metastases. SABR has also shown promising outcomes in patients with castration-resistant prostate cancer. [33].

## Conclusions

PSMA-PET/CT metastasis directed SABR provides excellent long-term efficacy in oligometastases of prostate cancer with a PSMA control rate of 86 % after 5 years. The decrease in PSMA expression is slow with the full effect of SABR observed sometimes not earlier than one year after treatment. The SABR regimen (35 Gy in five fractions) used for the treatment of the primary tumor is also effective in oligometastatic disease. Target volume definition based on PSMA-PET/CT imaging with minimal CTV-expansion resulted in excellent local control.

## CRediT authorship contribution statement

**Anna-Lena Zang:** Investigation, Writing – original draft, Visualization. **Timo Maier:** Investigation, Writing – review & editing. **Sandra Freitag-Wolf:** Formal analysis. **Alexander Fabian:** Writing – review & editing. **Severin Rodler:** Supervision. **Jürgen Dunst:** Writing – review & editing, Supervision. **David Krug:** Conceptualization, Methodology, Writing – review & editing, Supervision, Project administration. **Ulf Lützen:** Conceptualization, Writing – review & editing, Supervision, Project administration. **Olaf Wittenstein:** Conceptualization, Methodology, Writing – review & editing, Project administration.

## Declaration of competing interest

The authors declare the following financial interests/personal relationships which may be considered as potential competing interests: Alexander Fabian received honoraria from Merck Sharp & Dohme outside the field of this work. David Krug has received honoraria from Astra Zeneca, best practice onkologie, ESO, ESMO, Gilead, med update, Merck Sharp & Dohme, Novartis, onkowissen, and Pfizer, served on advisory boards for Gilead and has received institutional research funding from Stiftung Deutsche Krebshilfe and Merck KGaA. Other authors declare none.

## Appendix A Supplementary data

Supplementary Data 1 Example of contouring (a) and treatment plan (b) for PSMA-PET/CT guided SABR to a bone metastasis located in thoracic vertebra 7. GTV was defined based on the extent of Radiotracer uptake against PSMA with a GTV-to-CTV margin of 2-3 mm. CTV-PTV margin was additional 2-3mm. Fractionation regiment was 35 Gy in 5 fractions. *Abbreviations*. CTV=Clinical Target Volume; GTV=Gross Tumor Volume; PTV=Planning-Target-Volume. CT=Computer Tomography; PET=Positron-Emission-Tomography; PSMA=Prostate-Specific-Membrane-Antigen; SABR=Stereotactic Ablative Radiotherapy.
