# Supplementary figures and images for: PSMA response evaluation in follow-up PSMA-PET/CT after stereotactic ablative body radiotherapy (SABR) for oligometastases in prostate cancer
Source: Clin Transl Radiat Oncol. 2025 Jul 23;54:101021. doi: 10.1016/j.ctro.2025.101021 (PMC12328682; doi:10.1016/j.ctro.2025.101021)

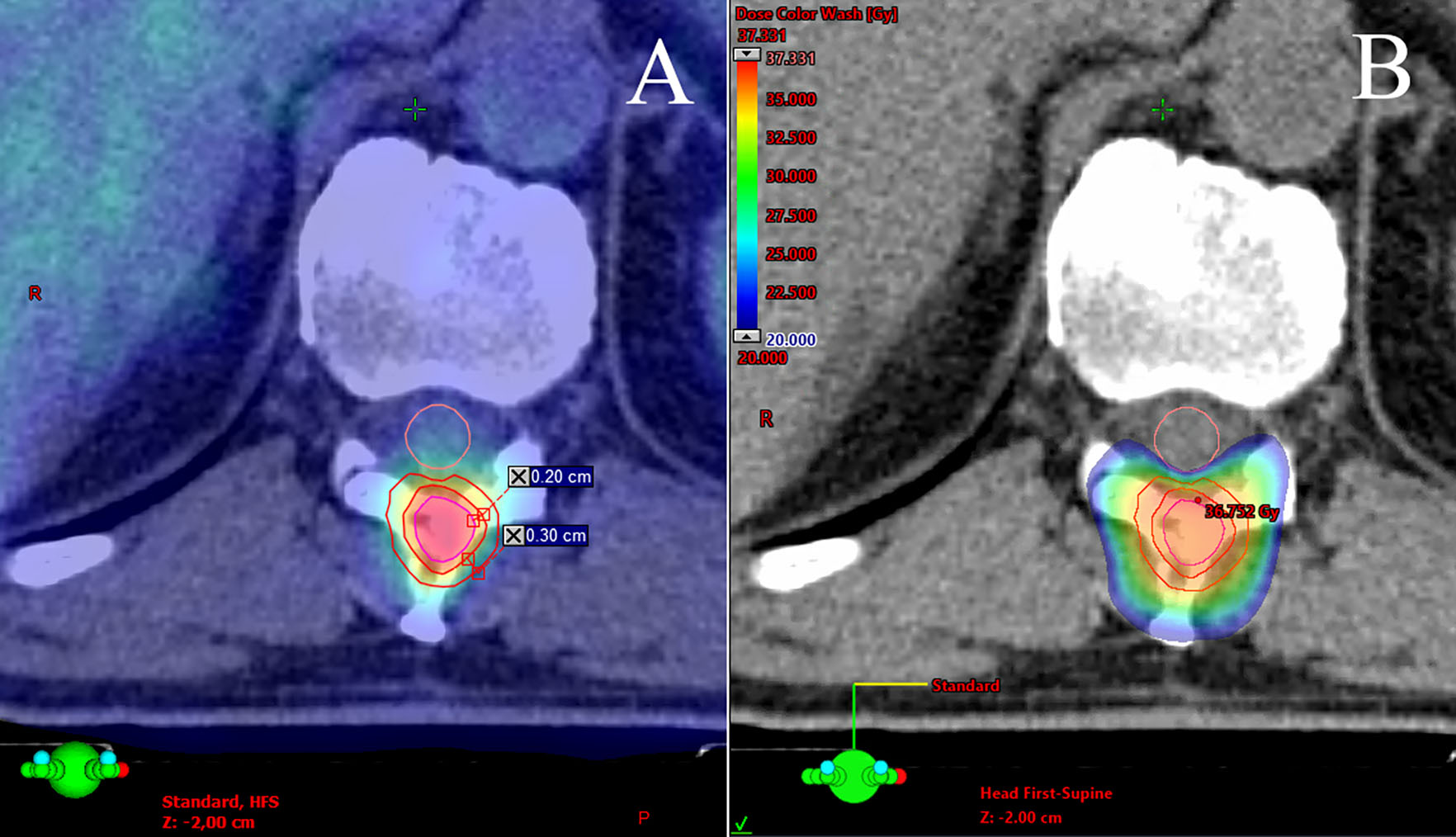

Supplement: Supplementary Data 1 — Example of contouring (a) and treatment plan (b) for PSMA-PET/CT guided SABR to a bone metastasis located in thoracic vertebra 7. GTV was defined based on the extent of Radiotracer uptake against PSMA with a GTV-to-CTV margin of 2-3 mm. CTV-PTV margin was additional 2-3mm. Fractionation regiment was 35 Gy in 5 fractions. Abbreviations. CTV=Clinical Target Volume; GTV=Gross Tumor Volume; PTV=Planning-Target-Volume. CT=Computer Tomography; PET=Positron-Emission-Tomography; PSMA=Prostate-Specific-Membrane-Antigen; SABR=Stereotactic Ablative Radiotherapy. [file mmc1.jpg]
